# Modernizing undergraduate medical education by bringing public health into focus

**DOI:** 10.3389/fpubh.2024.1451155

**Published:** 2024-09-11

**Authors:** Eleanor J. Hothersall

**Affiliations:** School of Medicine, University of Dundee, Dundee, United Kingdom

**Keywords:** medical education, curriculum design, population health, undergraduate education, social determinants of health

## Abstract

Healthcare and healthcare education are changing rapidly, being pulled in a number of directions by political, economic, environmental and technological imperatives. At the University of Dundee Medical School a curriculum review during the Covid-19 pandemic has allowed opportunities to reframe aspects of the program to emphasize themes linking public health and social determinants of health to the wider curriculum, while also bringing a shared understanding of the core outcomes of the program. This brings some adaptability into the structure and content of the course, helping graduates and staff to be “future proof.”

## 1 Introduction

Healthcare and healthcare education are at a time of significant flux and uncertainty, creating a rapidly changing landscape, and a need to create systems responsive to an unprecedented number of variables. Through all of this, Public Health (or Population Health) remains at the heart of healthcare. Rightly so: as the prevalence of non-communicable diseases rises inexorably there is a strong economic argument for preventive medicine, with even corporations understanding that there is more potential for profit from decades of healthy individuals compared with a shorter, less healthy life (1). There have been international calls for updates and changes to public health education (2–5). In a 2017 review of implementation of health promotion teaching in medical schools internationally, Hays commented that although public health increasingly features in the intended learning outcomes for medical graduates, how those topics are taught can be highly varied, and there is a lack of consensus of where the balance should lie between “awareness of concepts, knowledge about specific strategies and skills in implementing specific strategies” (6). This paper outlines how we are adapting an undergraduate medical program in Scotland to address the three main areas which are driving a need for change (changing technology, curriculum overload, and changes to the practice of medicine).

### 1.1 Changing technology

The Topol review (7) highlighted many ways that healthcare could be altered by technological improvements and innovations in the near future, but currently these changes have had comparatively little impact in healthcare, and so the ripples have not yet made their way back to education. There is considerable discussion about the impact that machine learning and artificial intelligence will have on provision of healthcare, but this is not yet taught in any detail.

Health promotion is similarly shifting from in-person delivery of services to virtual and asynchronous. The consequences for health inequalities are concerning. The most affluent can already have the experience of a cardiac disturbance highlighted by their smart watch, advising appropriate follow up (8). It was formerly science fiction to conceive of a society where most primary healthcare is delivered remotely or automatically, including behavior change, but the market in diet, exercise, and mental health support is already extensive. As inequalities in non-communicable conditions such as obesity (9) are widened by this differential access, so health inequalities will widen both within and between countries. Currently these concepts are not prominent in medical education curricula (10), but there is a clear need to include them.

### 1.2 Curriculum overload

Attempts to create overarching summaries of the medical curriculum have been most successful when summarizing a very abstract level of detail. For example, CanMEDs domains have titles such as *Leader*, *Health Advocate*, and *Professional* (11); in Pakistan the Standards for a Seven Star Doctor (12) include *Community Health Promoter*, *Professional*, and *Leader and Role Model*. Likewise, the UK’s General Medical Council highlights the need for *Professional Values and Behaviors* in medical students (13, 14). At the same time, the amount of factual knowledge a learner could potentially be expected to absorb increases daily—whether or not it is literally true that medical knowledge doubles every 73 days (15). To a learner, struggling with the “basics” of medicine, the prospect must be daunting indeed.

Meanwhile, the understandable desire for standardization, and accountability of our healthcare practitioners means that there is an increasing demand for standardized assessments and shared curricula. The implementation of the new Medical Licensing Assessment in the UK (16) for new graduates has generated debate about core content for assessment, and the validity or otherwise of these high stakes exams (17).

Medical training today still very closely resembles the experiences of doctors in training from the early 20th century. The emphasis on core science has a strong tendency to focus on cellular or even sub-cellular function and dysfunction, with decreasing emphasis the further one zooms out from that level of detail. For many years the need for doctors to understand interpersonal communication, including skills such as counseling, coaching, leadership and teamwork were completely overlooked by curriculum designers, concentrating on cramming as many facts as possible into the hours of teaching. This trend has been partially reversed (18, 19), and many medical schools now include “communication skills” within their curriculum (20), but this often still focuses on the one-to-one interaction between doctor and patient, rather than situating the doctor within a wider context. To avoid overloading the curriculum with excessive quantities of both facts, and concepts, medical education needs to start to move from “micro” to “macro.”

Meanwhile, innovation in the technology sector means that artificial intelligence and sophisticated algorithms may abruptly remove the need for what is currently core material (7). The challenge then will be to identify what is not needed, and how to identify the errors and biases inherent in the system. These are very different skills and paradigms from those normally emphasized when medical curricula are being developed (21). Until it is possible to predict which knowledge and/or skills will be unnecessary, educators continue to cram more and more in, while removing very little.

### 1.3 Changes to the practice of medicine

Political, environmental, financial and technological shifts in society mean that there are a wide range of priorities for healthcare and medicine, while the World Health Organization highlights an increasing deficit in healthcare providers worldwide (22). Students must attempt to navigate their way through these rapid shifts, to identify what is needed to enable them to progress through their current studies, but also what will be needed in the future.

At the same time as the role of the doctor is changing, so are the expectations and attitudes of the populations they serve. The recent Covid-19 pandemic brought challenges to those working in, or contemplating a career in, healthcare: at once it was apparent how much the population needs good healthcare provision, but also the level of frustration and antipathy expressed in some quarters caused many to question their role in society: is the demanding training and arduous work worth it in a world filled with skepticism and misinformation? This may be particularly pertinent in countries where remuneration of healthcare workers is comparatively low. Students experienced significant trauma during the pandemic, but made a significant contribution to the emergency workforce needed during this time (23, 24). In the long term, these experiences may themselves create dramatic changes to healthcare workers and systems.

To best serve the population, to adapt or design the best services and meet the needs of those we are charged with caring for, one needs high quality data, expert interpretation, and appropriate planning (25). To make decisions and proposals that are needs-based, and evidence-based rather than merely reactive or self-serving, those involved need a solid educational foundation which develops these skills, and enables doctors to articulate and actualize their values.

## 2 Context

The medical curriculum at the University of Dundee is designed to enable school leavers to gain a primary medical qualification and begin working as doctors in 5 years, in common with most medical courses in the United Kingdom. While some students may have a prior degree, or other qualification, many come directly from school, and due to the Scottish school system, can be as young as 17 when they first begin the course. Currently around 200 students matriculate onto the course each year. As the UK national health service (NHS) provides free healthcare at point of use, there is limited opportunity to increase student experience of healthcare through for example student-run free clinics (26). Almost 100% of graduates take up training roles in the NHS initially (27), though many migrate across the globe later in their careers. The main aim of the program is therefore to produce graduates who are ready for work as UK doctors from the point of graduation, with secondary aims of influencing later career development, including local retention and encouraging research activity.

Dundee has had an integrated curriculum since 1997, implemented by Harden and colleagues (28). Based on constructivist principles, the “spiral curriculum” allows students to revisit core learning over the years of their course, while also developing longitudinal themes which add enrichment and complexity to the experience. The primary focus is on competency-based education, with assessment through knowledge and clinical skill exams each year. The first 3 years of the course are highly structured, with a significant proportion of teaching in lecture and small group format, punctuated with clinical teaching to consolidate learning. The last 3 years of the course have an apprenticeship format, with students spending almost all of their time in clinical settings, gradually gaining confidence and skills, until ready to graduate as Foundation Year doctors.

While key themes such as communication skills and social medicine have been part of the curriculum from its initiation, the creation of Professionalism as a named component was a later evolution, made concrete by the UK General Medical Council’s release of the third iteration of their curriculum requirements, *Tomorrow’s Doctors* (29), in 2009. The most recent update of this document, now known as *Outcomes for Graduates* (13), lists key learning objectives relating to professionalism under the heading of “Professional Values and Behaviors.”

### 2.1 Curriculum review

At the University of Dundee medical school, we initiated a curriculum review immediately before the Covid-19 pandemic, so the content and implementation have both been necessarily affected by those events. In addition to a need to respond to the social changes outlined above, we wanted to adjust our curriculum to facilitate the transition for learners, particularly the increasing numbers coming from non-traditional medical backgrounds (“widening participation”). Additionally, we wanted to make changes to the structure of the course that would build a sense of community through educational continuity and small group working, that would support students in contextualizing their learning.

Using the double diamond design methodology from the Design Council (30), the core curriculum review team included the core academic leads for the program (experienced medical educators, coming from a range of medical disciplines including Public Health, Psychiatry, General Practice, Anesthesiology, Gynecology, Infectious Diseases, and Renal medicine), several medical students, and a number of colleagues from the Postgraduate Centre for Medical Education, including two of the School Associate Deans. Multiple stakeholder engagement events (including patient representatives, student groups and healthcare staff) were held both during the information gathering phase, and during the solution design phases, to ensure there was consensus across the school. Proposals were reviewed by a panel composed of experts from the medical school, other professional schools in the university, and external experts from other UK medical schools, and then later by the university periodic review process.

We identified twin strands of reform which we wanted to bring into the work. Both of these can be thought of as “future proofing,” however, one is public health facing, while the other seeks to develop adaptability in graduates, recognizing the rapidly changing healthcare environment contemporary graduates are likely to experience. Both are longitudinal strands, with representation in each year of the curriculum.

#### 2.1.1 The bigger picture

**Figure 1:**
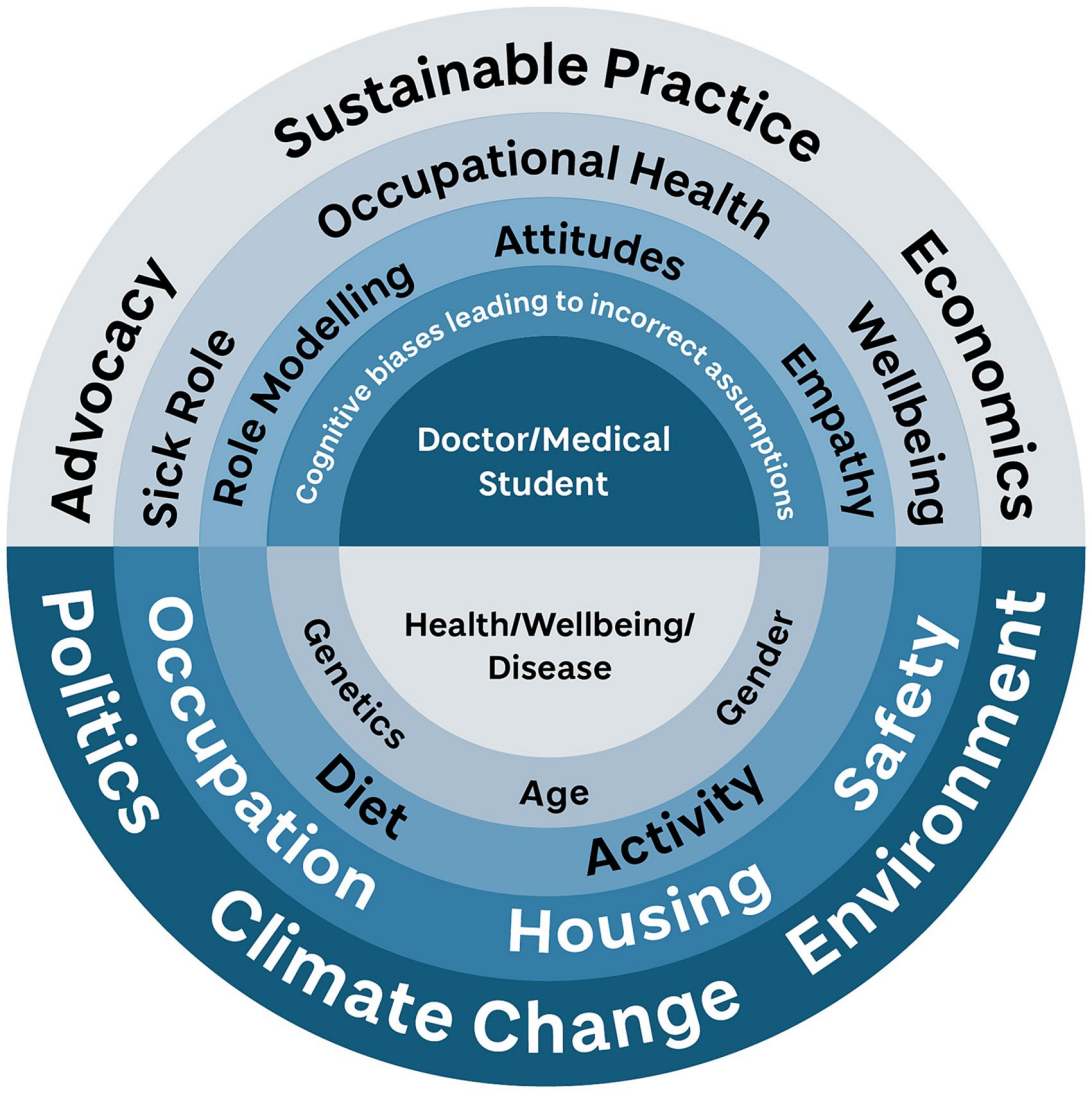
The core concept of the Bigger Picture elements of the Dundee curriculum. The student as healthcare professional is encouraged to see how all the layers of diagram influence the health and wellbeing of an individual and community, while at the same time seeing how they can affect and influence those layers. Designed by Eve Laws for University of Dundee, after Dahlgren and Whitehead (31).

This strand has a strong public health emphasis, with the explicit aim of bringing students to a greater awareness of both the social determinants of health (following models such as Dalgren and Whitehead (31)), and also the role of the doctor within society (see Figure 1). Ultimately the intention is for students to understand that each interaction they have as a healthcare provider (including when students) has wider ramifications, whether through antimicrobial stewardship thereby delaying the risk of multiresistant organisms (32), reducing inappropriate test requests, or pathologizing normal behaviors (33). The other significant dimension to enhance is the learner’s understanding of their role as an advocate for their patient and their population: by using their expertise and influence they can be powerful agents for change.

The Bigger Picture is introduced to students from the beginning of the course, with plenary sessions providing Scottish and local context for healthcare, and an explicit outline of the aims of the strand. Students also attend lectures, tutorials and workshops on related topics (particularly Healthcare Sustainability) within core teaching hours. These have not required additional hours in the syllabus as they have replaced previous didactic sessions in related areas, such as Public Health and Behavioral Sciences, but also some content which was previously taught elsewhere, such as a reflection on death and anatomy dissection is now part of this strand. By adding these additional insights to existing sessions, we provided deeper, contextual learning, a common challenge in teaching in this area (21). Additional teaching material is provided in elective components within the course, and opportunities for reflection on Bigger Picture topics. This is facilitated by a repository of learning materials designed mostly by students, outlining key areas they have identified as relevant, ranging from LGBTQ+ issues in healthcare, to social media and disinformation, to entrepreneurship and innovation, including artificial intelligence. We help students to link this learning back to other areas, particularly Quality Improvement, Public Health, and General Practice. At its core, the concept of the Bigger Picture is so wide-reaching that it is unrealistic and counter-productive to try to cover all of the individual areas of interest for each student. By following this model, we encourage students to identify areas of the Bigger Picture that are particularly resonant with them. By using their personal interests and experiences we encourage development of lifelong learning, and strengthen their reflective abilities.

#### 2.1.2 The Dundee Doctor

**Figure 2:**
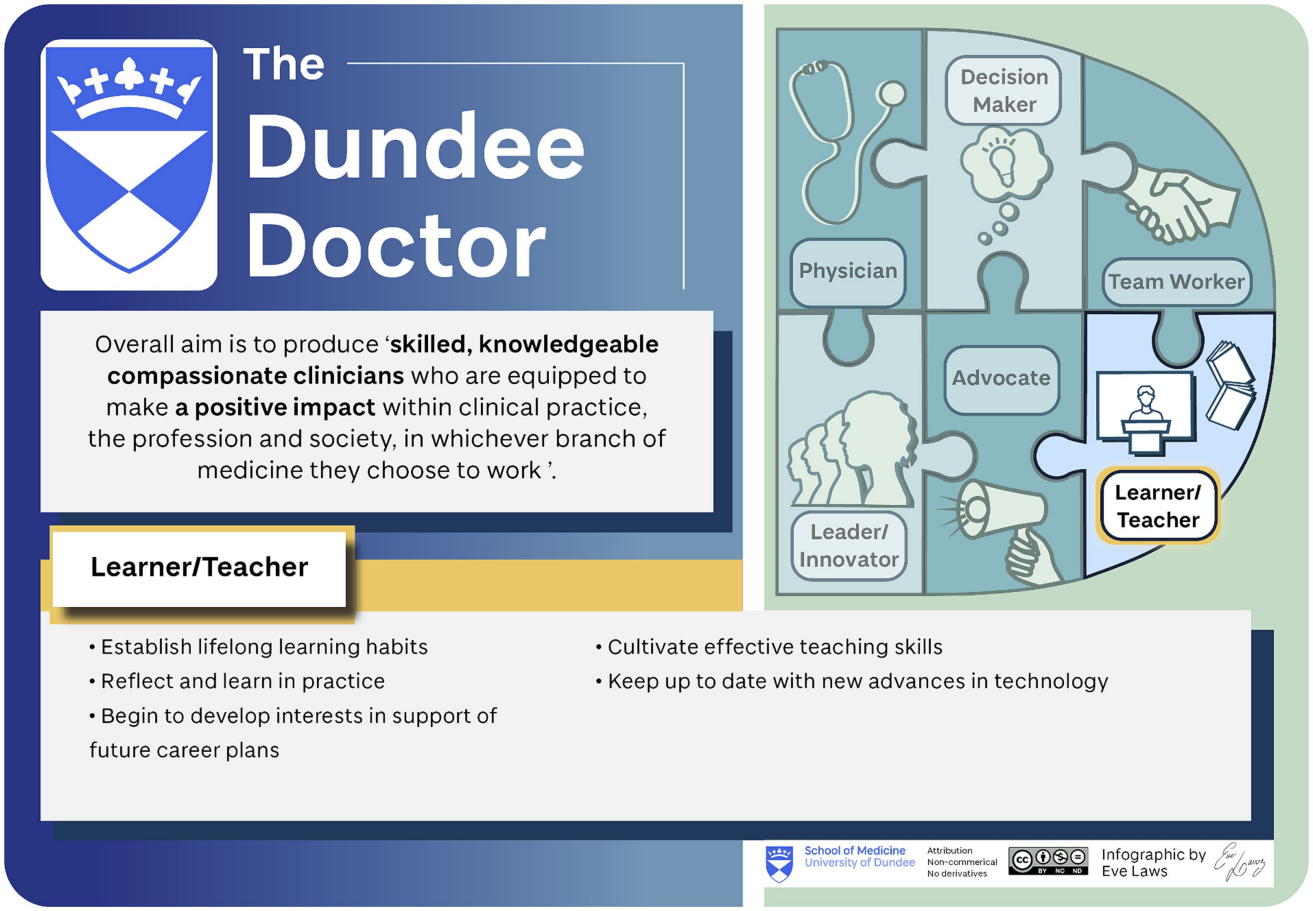
The Dundee Doctor. Designed by Eve Laws for University of Dundee.

The multiple stages of stakeholder engagement carried out during the information gathering phase of the curriculum review identified a common understanding of what makes a Dundee graduate unique. Where The Bigger Picture provides context for professional development, the Dundee Doctor has proved a valuable structure for curriculum implementation. The Dundee Doctor represents what may be lost if there is excessive “teaching to the test,” in light of the new national assessments (Figure 2).

#### 2.1.3 Assessment

Aspects of the Bigger Picture strand which are core taught material are included within the annual summative exam diet, however, more elements are within elective parts of the curriculum. Where students undertake a student selected component, these are normally four-week blocks with a summative assessment (for example an essay or presentation) at the end. Otherwise, Bigger Picture reflections are included in the students’ portfolio each year. These are reviewed but are not summatively assessed, until included within a final portfolio review assessment prior to graduation.

There is a significant challenge internationally to summatively assess these contextual learning outcomes (6)—in an era of increasing homogeneity in assessment via licensing exams, these more subtle outcomes need to be emphasized, for example through workplace based assessment, observed practice, or reflective portfolios, but these all have workload implications and it can be difficult to accurately assess these topics in short timescales (34). One reason this is difficult is because of the need to demonstrate a layer of application and process that goes deeper than knowledge acquisition (21, 35).

The Dundee Doctor does not have an assessed component specifically, but is rather used as a way of scrutinizing new proposals for taught content (“Is this consistent with the Dundee Doctor?”), and as a unifying vision for both staff and students.

## 3 Challenges faced

Implementing change to a curriculum as full and constrained as undergraduate medicine is enormously challenging. There is little margin for error due to the need for patient safety and the ongoing confidence of the public. The implementation of a new national exam means there is organizational anxiety about meeting those externally mediated academic objectives. Additionally, we knew that the previous program was well respected and produced excellent doctors. Consequently, every effort has been made to make the changes to the program as unobtrusive as possible, bringing reform through “quiet revolution” rather than disruption. This can be mistaken for inaction without a clear and consistent message from leaders.

The combination of core and elective elements of the new curriculum strands can be viewed as both a strength and a weakness of the course. If every element was core, there would be increased exposure and consistent coverage across the cohort. However, the topics covered are so wide ranging and diverse, it felt inappropriate to the curriculum design team to make all elements compulsory. Additionally, the amount of time that would take would be impractical given the other demands of the course. Through the Bigger Picture, learners have exposure to a wider range of content, allowing their curiosity and personal interest to guide them where they are most motivated. Allowing learners this opportunity may in turn directly improve academic achievement (36).

Formal evaluation of these curriculum elements has not been carried out at this time, at least in part due to the need to roll the changes out across three academic years, but this will be possible soon. Anecdotally student and staff have welcomed the changes, but student satisfaction may be a poor indicator of long-term impact, so a wider evaluation program is in development.

## 4 Discussion

It is posited that there is a decline in medical student empathy during the course of their studies (37). It may be that the current curricular emphasis on biomedical models of disease, and cure rather than prevention augments this. Certainly there is an overall decline in medical student understanding of social determinants of health during their studies (38, 39), persistent through decades of medical education, despite overt attempts to change this trajectory (40). Internationally, this area remains challenging, whether attempting to address antibiotic stewardship (41), climate change and environmental damage (42), lifestyle medicine (43, 44). Current approaches create overloaded curricula and do not create the adaptable, socially conscious doctors that are needed.

The introduction of new assistive technologies relating in particular to medical decision making and clinical reasoning, in the form of so-called artificial intelligence and machine learning emphasize the need for this adaptability. It is currently unclear what the role of the doctor will be in years to come, but we can be reasonably confident it will change rapidly, and significantly. We need to push our schools to develop programs that are “continuously renewing” (44), adapting to the future just like our students will. We have tried to bring that spirit to the new curriculum in Dundee, and look forward to the next steps.
